# Management for Patients with De Novo or Recurrent Tumors in the Residual Kidney after Surgery for Nonfamilial Bilateral Renal Cell Carcinoma

**DOI:** 10.1155/2009/135143

**Published:** 2009-11-30

**Authors:** Noboru Hara, Tsutomu Nishiyama, Norihiko Yoshimura, Satoshi Takaki, Kyoichiro Yamakado, Yasuo Kitamura, Kazuya Suzuki, Kota Takahashi

**Affiliations:** ^1^Division of Urology, Department of Regenerative and Transplant Medicine, Graduate School of Medical and Dental Sciences, Niigata University, Niigata 951-8510, Japan; ^2^Division of Radiation Oncology, Department of Molecular Genetics, Graduate School of Medical and Dental Sciences, Niigata University, Niigata 951-8510, Japan; ^3^Division of Radiology, Mie University Graduate School of Medicine, Mie 514-8507, Japan; ^4^Department of Urology, Niigata Cancer Center Hospital, Niigata 951-8566, Japan

## Abstract

The tumor de novo in the residual kidney after surgery for nonfamilial bilateral renal cell carcinoma (RCC) is problematic. We reviewed 5 patients who experienced such a situation. Three patients had had metachronous bilateral RCC, treated with radical nephrectomy in one kidney and nephron-sparing surgery (NSS) in the other. Two patients had had synchronous disease; one patient had received radical nephrectomy and NSS, and the other bilateral NSS. The 5 patients had another solid mass/de novo tumor in the residual kidney 16–88 (mean 46.8) months after surgery. For the tumor de novo in earlier years (1992–1999), one patient underwent surgery and hemodialysis, and the other selected a conservative observation. In recent years (2000–2007), one patient was conservatively observed; the remaining 2 received computerized-tomography-guided radiofrequency ablation, and the local tumors were well controlled postoperatively for 20 and 12 months with their renal function unimpaired. Ablative techniques can potentially strike a balance between oncological and nephrological outcomes in patients with sporadic multiple RCC, successful management of which was difficult previously.

## 1. Introduction

Bilateral/multiple renal cell carcinoma (RCC) is commonly associated with hereditary disorders represented by von Hippel-Lindau disease, a well-known familial syndrome; recently, RCC in patients with von Hippel-Lindau disease has been treated with well-planned, sequential nephron-sparing approaches, since metachronous multiple occurrences of RCC can be predicted at the initial diagnosis [1]. On the other hand, sporadic/nonfamilial bilateral RCC is infrequently encountered, and its management is also problematical; prediction of the clinical presentations such as postoperative de novo occurrence or recurrence of disease is impossible in sporadic cases [2, 3]. Recurrence of renal tumors in patients who have received surgery for sporadic bilateral RCC represents a serious situation in the era of nephron-sparing surgery/partial nephrectomy, because it is difficult to strike a balance between oncological and nephrological outcomes in treating such cases. Yet, their clinical course, management, and outcome have not been studied thus far. We reviewed 5 patients who had de novo or recurrent renal lesions following surgery for metachronous or synchronous bilateral RCC without any familial history and associated syndrome, and reported their oncological and nephrological outcomes to underscore the clinical presentation and transition of intervention. We therein described 2 patients recently treated with computerized-tomography-guided percutaneous radiofrequency ablation therapy (RFA) for renal tumors emerging in the residual kidney after surgery for sporadic bilateral RCC. 

## 2. Patients and Methods

We reviewed the clinical and pathological record between January 1992 and December 2007 in the Department of Urology, Niigata University Hospital, and associate institutions. Five patients were found with renal masses in the residual kidney following surgery for sporadic/nonfamilial bilateral RCC. These masses were solid on CT, and were thought most probably de novo or recurrent RCCs. Clinical and pathological stages were determined according to the International Union Against Cancer (UICC) classification of 2002 [4]; for cases in earlier years, those were reassessed using this criterion. Clinical staging routinely included chest radiograph and abdominal computerized tomography (CT). All subjects for clinical interventions gave informed consent to all patients. Informed consent to use the data for clinical or basic researches was obtained from all the patients. 

The procedure for the patients treated with RFA was approved by a suitably constituted Ethics Committee of Niigata University Hospital. RFA was performed with previously reported devices and techniques [5]. Briefly, prophylactic antimicrobials were administered 1 hour before treatment. An RF generator (Cool-tip Radiofrequency Ablation System, Radionics, Burlington, Mass, USA) was used under local anesthesia and sedation with intravenous phentanyl citrate. The single cool-tip RF electrode was placed with a real-time CT-guided method, referring to the tumor size, shape, and localization to ablate whole tumor tissue. The proximal margin of the tumor was initially ablated to achieve sufficient ablation of the deeper central portion of the kidney, and superficial treatment was subsequently performed. The target probe temperature was rendered at 100°C. Tumors were heated at 65°C in a 12 minute cycle with a maximum electrical power of 50 W, and a second RF cycle was applied when the tissue temperature could not be adequately maintained. After the probe was withdrawn, RF energy was additionally given to the intraparenchymal and perirenal needle tracks to minimize bleeding and tumor cell dissemination. Dietary intake was started 3 hours after treatment, and limitations of activity were lifted on the next day. Follow-up CT was performed every 3-4 months for the initial 12 months and thereafter every 6 months.

## 3. Results

Patients and their characteristics are shown in Table 1. The clinical presentation of these patients is summarized as follows: patients' age ranged between 42 and 62 (mean 53.4) years at the initial visit. One patient was female, and the remaining 4 were male. Three patients were asymptomatic, and the tumors were incidentally found on abdominal ultrasonography or CT performed for other purposes. Two patients were symptomatic, and presented with hematuria and/or pyrexia. Two patients had synchronous bilateral disease, and 3 had a metachronous tumor 4, 7, and 11 years after primary radical nephrectomy. Of the 2 patients with synchronous disease, one patient had T1a in both sides and N0M0 disease, and one had T1b and T1aN0M0 disease. In 3 patients with metachronous bilateral RCC, the primary disease was T1bN0M0, T2N0M0, and T1aN0M0, respectively; all of secondary tumors were diagnosed as T1a. The patients were followed postoperatively with chest roentgenogram/thoracic CT and abdominal CT every 6 months for the initial 3-4 years and thereafter annually when they did not have a recurrence.

 The clinical course and outcome of the 5 patients are presented in Table 2. Of the 2 patients with synchronous bilateral disease, one patient underwent first radical nephrectomy for the tumor in one kidney and subsequently nephron-sparing surgery for the other kidney; the other patient was treated with nephron-sparing surgery/partial nephrectomy for both kidneys in separate sessions. The 3 patients with metachronous diseases received radical nephrectomy for the primary disease, and the metachronously appearing tumor in the contralateral kidney was treated with nephron-sparing surgery. The pathological diagnoses of the 10 tumors were conventional RCC G2, G2-dominant with G1, or G2-dominant with G3. Surgical margins and lymph nodes were pathologically negative for cancer cells in all the specimens.

**Figure 1:**
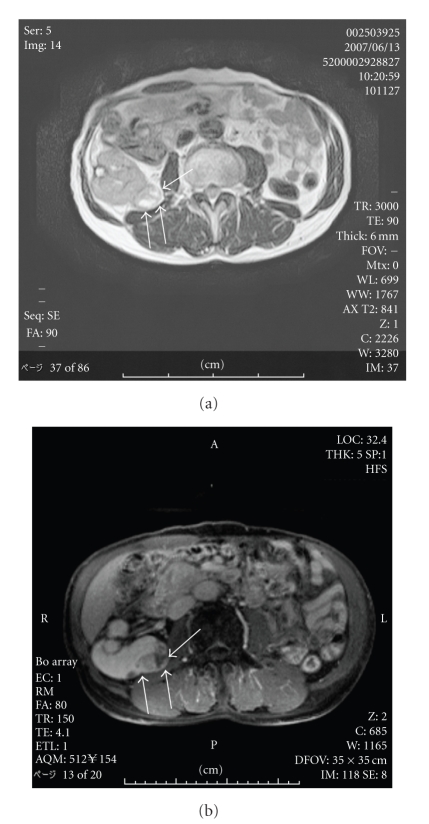
(a) T1-weighted magnetic resonance imaging showed a solid mass measuring 3 cm with heterogeneous intensity in the residual right kidney (case number 4). (b) The local tumor was well-controlled for 20 months after CT-guided RFA.

**Figure 2:**
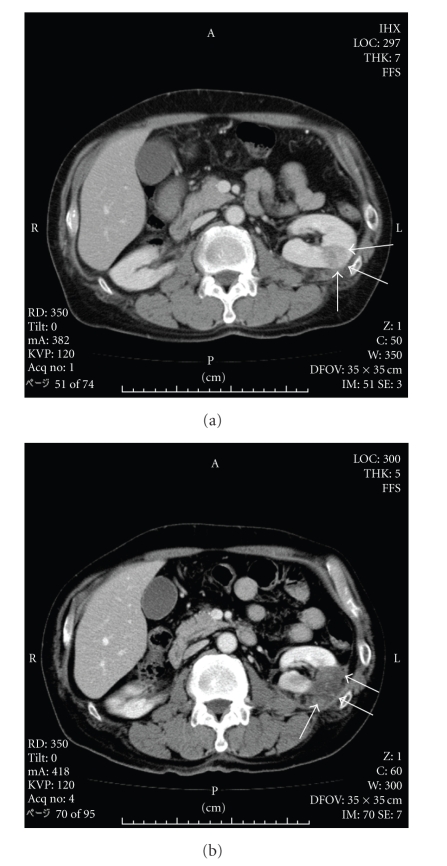
(a) Contrast-enhanced CT showed a solid tumor of 2.5 cm in diameter with weak enhancement in the left kidney (case number 5). (b) The tumor was replaced by low-density tissue without enhancement following RFA, and he was free of disease postoperatively for 12 months.

The 5 patients had a recurrent solid mass in the residual kidney on CT during the follow-up period for 16–88 (mean 46.8) months after last surgery, which was thought most probably metachronous de novo or recurrent RCC (Table 2). Of the 2 patients having a de novo tumor in earlier years (1992–1999), one patient underwent excision of the residual kidney, resulting in a dialysis-dependent status, and the tumor was histologically confirmed with RCC G2 (case number 1 in Tables 1 and 2). The other patient was managed with a conservative observation according to informed consent (case number 2 in Tables). Although her course was lost 29 months after recurrence in the residual kidney, the tumor gradually enlarged during the observation period (2.0 cm to 3.0 cm). Of the 3 patients in recent years (2000–2007), one patient selected the conservative management, and was alive with disease thereafter for 22 months (case number 3 in Tables); his tumor slightly enlarged during the observation. The remaining 2 patients (case number 4 and case number 5 in the tables) underwent CT-guided RFA, and their courses are summarized as follows; the former patient (case number 4) underwent radical nephrectomy for a nonmetastatic T1b left renal tumor, pathologically diagnosed as conventional RCC G2, when he was 62 years old. Eleven years after surgery, he received nephron-sparing surgery for a small right renal tumor with same pathology. Sixteen months after the second operation, a metastatic pancreatic lesion was treated with surgery. It was pathologically diagnosed with RCC G2-dominant with G3, and during the follow-up period, a renal tumor most probably RCC measuring 3 cm was found in the remaining right kidney (Figure 1(a)). Because of his poor renal function (serum creatinine level of 2.2 mg/dL, creatinine clearance of 28 mL/min) and insecure prognosis, the tumor was treated with RFA. The local tumor shrank and did not enlarge postoperatively for 20 months with his renal function preserved (serum creatinine level of 2.3 mg/dL) (Figure 1(b)), although a new metastatic lesion was found in the pancreatic head. The latter patient (case number 5) was diagnosed as having bilateral T1a renal tumors, when he was 42. Both tumors were managed by nephron-sparing surgery, and were pathologically diagnosed as RCC G2. Eighty-eight months after surgery, a solid renal tumor of 2.5 cm in size arose from the parenchyma center of the left kidney (Figure 2(a)). The patient was also included in criteria of chronic kidney disease with a serum creatinine level of 1.5 mg/dL (creatinine clearance of 36 mL/min). Also, he had poorly controlled epilepsy and severe alcoholic liver injury, and received RFA for the de novo renal tumor emerging metachronously. He was free of disease with no evidence of local recurrence or metastases postoperatively for 12 months (Figure 2(b)), and his renal function was well preserved (serum creatinine level of 1.4 mg/dL).

## 4. Discussion

The present report showed practice of the clinical course and management in patients with de novo or recurrent renal neoplasm in the residual kidney, who had received surgery for sporadic bilateral RCC. Their disease history was very long, and it is thought impossible to predict metachronous occurrence of another renal lesion. Becker et al. reported the long-term outcome in 101 patients with bilateral RCC, and showed relatively favorable prognosis in them (probability of the 5-, 10-, and 20-year cause-specific survival rate was 91.9%, 79.1%, and 56.7%, resp.) [6]. Also, 57.4% of these patients had metachronous occurrences, and they were significantly younger than those with synchronous disease at diagnosis (median age 53.6 versus 58.7 years, *P* < .05). Although no patient in their series was described with de novo occurrence in the residual kidney after bilateral surgery, more frequent incidence and younger age in metachronous cases may suggest the need for treatment options in patients under such situation as featured in the present report.

In the current report, one patient in earlier years received excision of the residual kidney, and required hemodialysis. Two patients did not accept hemodialysis, and selected the conservative management. Thus, nephron-sparing as well as oncological disease control is a matter of concern also in the patient group featured herein. Hoffmann and associates reported short-to-intermediate-term outcomes of patients with RCC arising from a solitary kidney after nephrectomy, who were treated with RFA [7]. All 10 patients in their study had a history of nephrectomy, and the recurred tumor size in the contralateral kidney ranged between 1.9 and 4.2 cm (mean 2.7 cm). No tumor recurrence or major complication was observed postoperatively for 3–24 months, and none of these patients became dialysis-dependent. In their series, one patient developed another RCC and was successfully treated with second RFA. Their data and our results suggest that less invasive, nephron-sparing techniques such as RFA or cryotherapy can preserve renal function without compromising the oncological efficacy, and RFA may be a feasible option for patients with a de novo renal tumor in the residual kidney, who have received prior surgery for sporadic bilateral RCC. However, RFA for such cases has several limitations. First, the therapeutic efficacy of RFA is assessed by radiographic findings, and it is impossible to study pathological margin [8]. Second, RFA was undertaken without biopsy for safer treatment, because the 2 patients were highly complicated both anatomically and functionally in kidney conditions, and also the imaging findings, clinical course, and previous pathology strongly suggested presence RCC in the residual kidney. Some previous studies have suggested limitations in the efficacy of biopsy followed by ablative techniques [5, 7, 8]. Third, long-term oncological outcomes are needed to draw a definite conclusion for the exact utility of RFA in such patients.

In summary, we reported clinical practice in patients with de novo or recurrent renal neoplasm, who had received surgery for sporadic bilateral RCC. RFA could preserve renal function without compromising the short-to-intermediate-term oncological outcome, and may be a feasible option for such patients, although further studies on the assessment for its therapeutic efficacy and biopsy technique are warranted.

## Figures and Tables

**Table 1:** Patients, their characteristics at the initial visit, occurrence pattern of renal tumors, and surgical approaches.

Patient number	Age (y.o.)	Sex	Leading symptom	Stage	Occurrence pattern	Nephrectomy
Case 1	48	male	hematuria	T1b, T1a	synchronous	radical and partial
Case 2	54	female	pyrexia	T2, T1a	metachronous	radical and partial
Case 3	61	male	none	T1a, T1a	metachronous	partial and partial
Case 4	62	male	none	T1b, T1a	metachronous	radical and partial
Case 5	42	male	none	T1a, T1a	synchronous	partial and partial

All the patients had N0M0 disease at the initial visit or when the metachronous tumor emerged in the contralateral kidney. All the tumors were pathologically diagnosed as conventional renal cell carcinoma (RCC) G2 or G2-dominant, and surgical margins in patients receiving partial nephrectomy were negative for malignant tissue.

**Table 2:** Clinical course and outcome of the patients.

Patient number	Disease-free duration (months)	Year of reoccurrence	Management for reoccurrence	Oncological outcome	Nephrological outcome
Case 1	33	1992	nephrectomy	Disease-free for longer than 10 years	Dialysis dependent
Case 2	41	1995	Conservative observation	Lost 29 months after reoccurence	Dialysis-free
Case 3	56	2005	Conservative observation	Alive with disease for 22 months	Dialysis-free
Case 4	16	2006	RFA	Alive with metastasis for 20 months	Dialysis-free
Case 5	88	2007	RFA	Disease-free for 12 months	Dialysis-free

Case number corresponds to that of Table 1. All the de novo or recurrent tumors in the residual kidney (reoccurrence) were diagnosed as T1a disease on computerized tomography (CT). RFA, CT-guided radiofrequency ablation.

## Abbreviations

RCC: Renal cell carcinoma
RFA: Radiofrequency ablation
CT: Computerized tomography
